# Translating *in silico* micro-computed tomography bone scans into a 3D printable bone model

**DOI:** 10.3389/fbioe.2026.1836815

**Published:** 2026-09-11

**Authors:** Lucy Dascombe, David A. Chorlton, Tim Nichol, Christine L. Le Maitre, Nicola Aberdein

**Affiliations:** 1 Biomolecular Sciences Research Centre, Sheffield Hallam University, Sheffield, United Kingdom; 2 Department of Physics and Astronomy, University of Manchester, Manchester, United Kingdom; 3 School of Medicine and Population Health, University of Sheffield, Sheffield, United Kingdom

**Keywords:** bone, computer-assisted design, micro-computational tomography, three-dimensional model, three-dimensional printing

## Abstract

Personalised medicine is an expanding area of medical research, in which patient-specific physiology informs clinical interventions. The personalisation of skeletal-based medical implants through 3D imaging and additive manufacturing (AM) has the potential to improve patient outcomes and reduce the likelihood of implant rejections. Currently, full-body computed tomography (CT) scanners obtain images at the limit of resolution of intricate internal bone architecture such as trabecular bone (Tb), which makes them difficult to map with precision in humans. Therefore, we have proposed the development of a 3D printing workflow which utilises scaled high-resolution micro-CT outputs that maintain bone topography in the final 3D print, specifically the retention of physiologically relevant trabeculae. In this study, we evaluate 3D model generation and rendering parameters that are necessary to maintain anatomical fidelity while ensuring printability. Meshing algorithm, computer-aided design (CAD) rendering tools, and the resulting surface tessellation of *ex vivo* bone samples were evaluated to identify computational limitations and their impact on downstream fabrication. A proof-of-concept workflow was developed, in which high-resolution micro-CT datasets preserving intact Tb morphology were successfully scaled and translated into anatomically informed 3D-printed models using fused deposition modelling (FDM) and stereolithography (SLA).

## 1 Introduction

Within the field of osteo-specific research, many different experimental approaches have been used for the generation of three-dimensional (3D) bone models, particularly within the prosthetics field (Qu et al., 2024; Qu et al., 2024; Qu et al., 2024). Current 3D model workflows typically rely on clinical computed tomography (CT) to reconstruct the external morphology of bone for additive manufacturing (AM); however, these approaches generally cannot preserve micro-scale architecture during fabrication and often rely on bone regeneration after implantation for clinical translation (Cao et al., 2021; Nyirjesy et al., 2022; Palmquist et al., 2023). In particular, trabecular bone (Tb) is often represented as a porous lattice structure to encourage osseointegration, with mixed success (Fraser et al., 2024; Pu et al., 2022). While advances in imaging and AM have enabled increasingly sophisticated anatomical models (Qu et al., 2024; Qu et al., 2024; Qu et al., 2024), translating high-resolution biological datasets into physiologically representative printable models remains a challenge. Each stage of the workflow, from mesh generation and computer-assisted design (CAD) rendering to slicing and fabrication, influence the anatomical fidelity, computational efficiency, and printability.

### 1.1 3D workflow priorities

3D medical imaging techniques, each with distinct spatial resolution limitations, enable the creation of replicas of biological structures using CAD without the requirement for manual modelling. These modalities include clinical CT, micro-CT, and nano-CT, which operate across approximate resolution ranges of ∼300 μm–700 μm, 1 μm–50 μm, and 50 nm–500 nm, respectively (Ohs et al., 2020; Tang et al., 2024), along with dual-energy X-ray absorptiometry and magnetic resonance imaging (Mandolini et al., 2022; Virzì et al., 2020). While clinical CT is capable of capturing the overall bone morphology, it cannot accurately resolve the hierarchical organisation of bone due to the fundamental resolution gap. This limitation affects the representation of key structural features, including the cortico-trabecular interface, Tb morphometry, and surface topography, often necessitating computational reconstruction approaches or specialised high-resolution scanners (Frazer et al., 2024; Oláh et al., 2022; Tang et al., 2024). In contrast, micro-CT enables high-resolution imaging of smaller bone specimens such as those from small rodent models, which provide detailed volumetric data and accurate characterisation of Tb architecture (Tang et al., 2024). However, the possibility of reverse engineering these small rodent model datasets so they can be scaled and translated into high-fidelity 3D models has not been fully evaluated.

**FIGURE 1:**
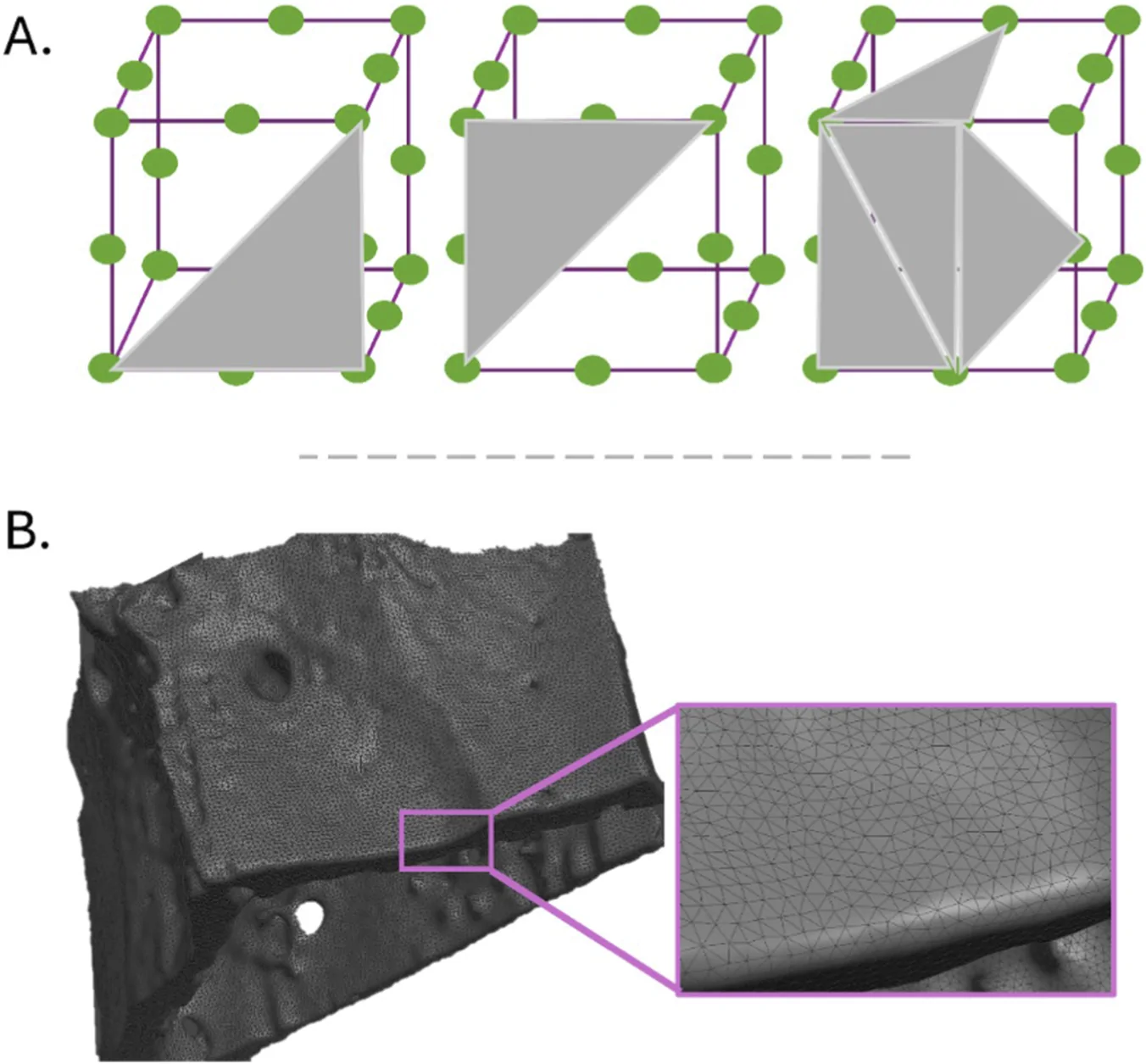
Topological isoform configurations of Marching Cubes 33 meshing algorithms and the resulting tessellated mesh wireframe on a mouse vertebral body (VB). **(A)** Marching Cubes 33, double times cubed, and adaptive rendering all follow the same principal of mesh generation but differ with the volume of information, accuracy, and quantity of empty cell reads. Mesh is defined by both vertices (green) and triangles (grey), which are combined to form 3D information. **(B)** Adaptive rendering tessellated wireframe on mouse VB rendered surface, with zone of artificial magnification (purple).

Reconstructed micro-CT projections can be exported in a variety of file types, such as .stl and polygon file format (Ply) for digital imaging and communications in medicine (Flaxman et al., 2021; Kamio et al., 2020), dependent on the imaging and reconstruction system (He et al., 2019; Steiner et al., 2020). To generate a 3D model, the entire object surface requires a triangulated mesh boundary. Different meshing algorithms introduce various compromises between geometric fidelity, computational complexity, and downstream manufacturability. By generating a mesh, subdivision of continuous geometric domains are produced to provide information on surface structure, tolerance, and boundaries (Figure 1) (He et al., 2019; Lee et al., 2020). Structured or mapped mesh follows three tessellation rules: no overlapping or gaps, tiles must be regular consistent polygons, and each vertex must look identical. The algorithm is complex as the geometrical calculations are completed in a two-dimensional (2D) space; however, this can lead to errors when the mesh is translated into a 3D geometry. Unstructured or direct meshing is the alternative, with vertices connected in irregular patterns for more complex shapes and surfaces. However, this method tends to produce a higher frequency of meshing errors as the algorithm attempts to mesh the entire 3D geometry without sub-dividing it into 2D reading planes (Wang et al., 2017; Yu et al., 2022).

3D model outputs vary depending on the selection of the triangle meshing algorithm (Figure 1A) applied to the reconstructed 2D image stack generated during micro-CT acquisition. Common meshing algorithms include Marching Cubes 33 (MC33), double times cubed (DTC), and adaptive rendering (ATR). MC33 is an algorithm to extract 2D surface mesh from a 3D volume by iterating across the volume of interest (VOI), searching for regions that cross the level of interest to create a uniform mesh across the boundary of information. The entire VOI is split into empty cells, and if the cells contain voxel information, it is compiled into a 2D mesh with neighbouring voxels (Figure 1A), which is then filtered into a 3D polygonal mesh model to represent the triangular surface according to standard meshing rules (Figure 1B) (Custodio et al., 2019; Lee et al., 2020). DTC is similar to MC33; however, fewer regions are read as actions; therefore, it produces a smaller file size and reduced surface detail (Botha and Charl, 2013). Adaptive rendering is an algorithm designed by the manufacturer Bruker Ltd, and the mesh characteristics follow meshing rules, like MC33 and DTC, but with higher control. For example, reduced locality, reduced noise between detected pixels, and tolerance-defined accuracy between pixel borders to assist in a successful mesh generation with minimal boundary errors.

Despite the choice of algorithm, different 3D model file types can be exported. This includes .stl, Ply, connect textures, and polygon-trochoid-profiles. Both .stl and Ply are widely-accepted mesh file types, whereas connect textures and polygon-trochoid-profiles are Bruker-specific file types. As the most widely adopted format for AM, .stl was the selected file type for the workflow presented here; therefore, the methodology reported here will utilise this file type (Kamio et al., 2020). Models can be exported as either ASCII or binary, with ASCII encoding coordinates of the vertices of each triangle in the exact location producing a highly complex 3D model, while binary encodes the components of unit vectors normalised to each triangle (Isenburg and Snoeyink, 2002). Finally, the 3D model can be exported in different units (μm, voxel, mm, and inch) to alter the overall dimension of the model in proportion to the original scanning resolution.

This proof-of-concept method aims to evaluate the workflow required to scale physiologically accurate Tb architecture from a small rodent model into a 3D-printed bone model. We aim to demonstrate that scaling high-resolution micro-CT datasets that retain Tb architecture beyond the resolution limitations of conventional clinical CT can produce a physiologically accurate model in the cm range (Burghardt et al., 2011; Klintström et al., 2022).

## 2 Materials and methods

### 2.1 Experimental design and rationale

**FIGURE 2:**
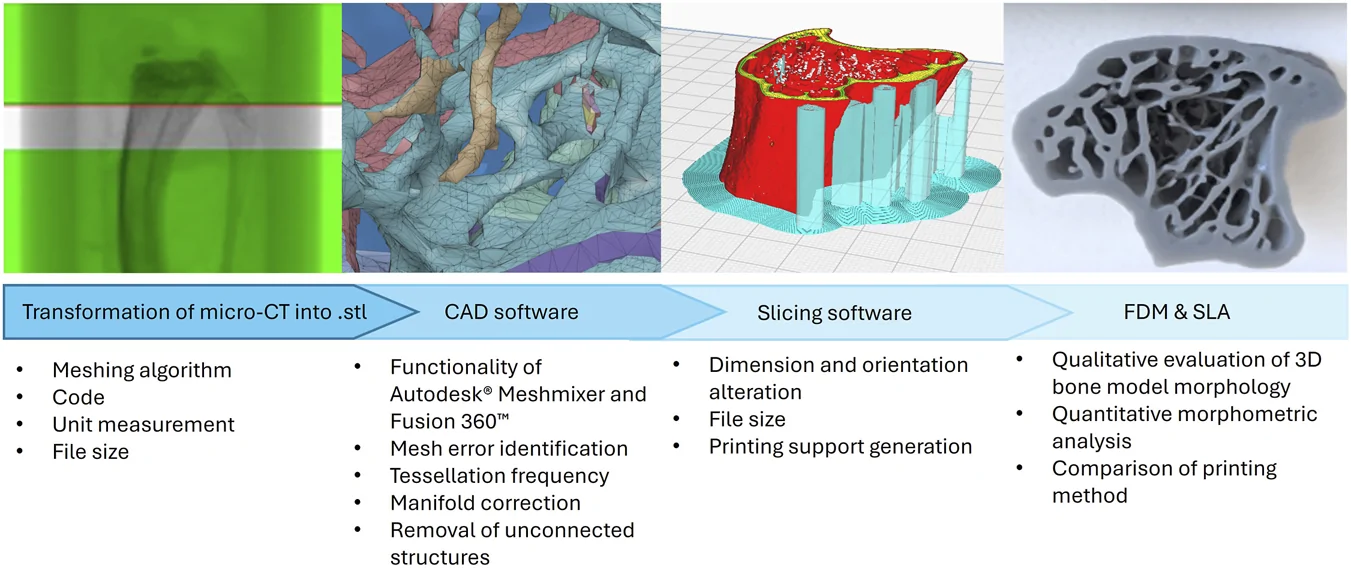
Workflow schematic illustrating the key stages for transforming micro-CT *ex vivo* bone into a 3D-printable bone model, highlighting the parameters explored at each stage.

High-resolution micro-CT datasets of murine bone morphometry were acquired and transformed into .stl models. Various meshing algorithms, export codes, and units of measurement were evaluated, with file size and 3D tessellation errors analysed. The optimisation criteria were predefined to identify an .stl export condition that balanced preservation of bone morphology with downstream manufacturability. Candidate export settings were evaluated according to the acceptable file size within hardware and software limitations; minimal mesh errors; preservation of anatomical morphology; and compatibility with subsequent CAD processing, slicing, and successful 3D printing. The resulting 3D models were rendered in two computer-aided design software programs, Autodesk® Meshmixer and Autodesk® Fusion 360™, to examine the effect on tessellation and the preservation of complex morphology. Finally, the rendered 3D bone models were sliced, with structural supports configured according to the requirements of FDM and SLA manufacturing (Figure 2). The rationale of this proof-of-concept methodology was to evaluate how computational decisions made throughout the workflow influences the balance between preservation of bone microarchitecture and downstream manufacturability.

### 2.2 Tissue collection

Mixed sex ex-breeder Sprague Dawley rats (N = 6) aged 26 ± 1 weeks and fed a normal diet were obtained *postmortem* from the University of Sheffield. Rats were sacrificed using schedule-1 certified method by cervical dislocation with prior use of sedative followed by permanent cessation of the circulation to confirm death. Male C57 mice (N = 3) aged 24 ± 2 weeks and fed a normal chow diet were collected as previously described (Williamson et al., 2023). Samples were collected *via* whole-body dissection to isolate the tibia, femur, and vertebral bones and fixed in 4% paraformaldehyde for 36 h–48 h at 4 °C before being transferred to 70% v/v ethanol for storage until experimentation.

### 2.3 Micro-CT imaging and processing

Mouse and rat right tibiae, right femora, and lumbar three (L3) vertebrae were scanned using micro-CT (SkyScan 1272, Bruker Micro-CT, Belgium) with a resolution of 9-μm voxels by wrapping the bone in moistened tissue paper and mounted within a plastic scanning tube and secured onto the micro-CT stage. Samples were scanned using a 0.5-mm aluminium filter for mouse samples and a 1-mm aluminium filter for rat samples. A 2,016 × 1,344 camera detector was used along with an X-ray source current set to 55 kV:160 μA for mouse samples and 65 kV:140 μA for rat samples. Flat-field detector calibration was performed before scanning to minimise beam hardening and improve the signal-to-noise ratio by reaching optimum X-ray attenuation. Three X-ray projections were acquired every 0.3°–0.5° rotation steps. Samples were scanned over a full 360° rotation.

X-ray outputs were reconstructed from 2D to 3D file format using NRecon (NRecon 2.1.0.2, Bruker Micro-CT, Belgium). Reconstructed images were optimised using automatic post-alignment compensation, +1 smoothing filter (Gaussian window kernel), 40% beam-hardening correction, and appropriate ring artefact correction. A threshold of 0 mm^-1^–0.12 mm^-1^ was selected to represent the transition between pore and bone tissue for all rat and mouse samples. To generate a representative VOI from each full bone sample, CTAn (1.21.2.0) was used to segment a region that contained both Tb and cortical bone (Cb). To ensure consistency, the VOI was selected using the bridge-break of the growth plate as a suitable and consistent landmark for tibia and femur and cancellous bone for the vertebra body (VB) after isolation of processors and pedicles. Each VOI was a total height of 2 mm for rats and 1.5 mm for mice.

To assess the difference between meshing algorithms, code, and unit, n = 3 mouse tibia were exported in every variation of the meshing algorithm (MC33, DTC, and ATR), in ASCII or binary code, and unit (μm, voxel, mm, and inch) as .stl files. All .stl visualisation, processing, and rendering were completed on a Windows 10 Enterprise PC system, with 16 GB RAM and an Intel® Core™ i5-8500 CPU @ 3 GHz. Models were uploaded to Autodesk® (San Francisco, California) Meshmixer (version 11.0.544) to visualise errors, tessellation total, and dimensions. Based on the predefined optimisation criteria, ATR with +1 smoothing, locality 1.00, tolerance 1.00, binary encoding, and mm were the selected conditions for the generation of the .stl model to progress with rendering and different 3D printing techniques. For the final rendered .stl models, the median bone of all bone morphometry values was selected as a representative bone model.

### 2.4 STL rendering

Rendering was completed in both Autodesk® Meshmixer and Autodesk® Fusion 360™ to assess the difference in .stl output. In Autodesk® Meshmixer, basic rendering was completed to both identify and eliminate errors, re-size the model, and ensure the 3D model was manifold. The mesh was repaired for errors by uploading the .stl model into the interface, selecting analysis, followed by the inspector. Errors in the mesh were repaired using the hole fill mode with the smooth fill option. The 3D printer stage and upper limits were input into the software, with the model fixed to the build plate. Finally, the model was made manifold using the edit feature to ensure the tessellated surface was free from breakages. Make solid was selected, with the accurate type selected to minimise changes to detail. Once rendering was completed, the model was exported as an .stl file.

Basic rendering in Autodesk® Fusion 360™ replicated the initial workflow performed in Autodesk® Meshmixer prior to more advanced tessellation control. For basic rendering, the model was imported into the design workspace, where tessellation frequency was reduced by 20% using the mesh editing feature. The modify tab allowed proportional editing of the tessellation by reducing and re-meshing. The mesh was then repaired using the stitch and fix function before exporting the basic .stl file. For complex rendering, face groups were generated with fast type selected, enabling individual connected mesh components to be visualised and inspected. Face groups allowed isolated Tb structures that were not physically connected to the main Cb body or Tb network to be identified. A small number of isolated Tb isoforms were manually removed following inspection from multiple orientations, as they were hypothesised to increase the probability of print failure by generating unsupported features during physical fabrication. This decision represented a deliberate compromise between preserving anatomical fidelity and ensuring successful manufacture, whilst retaining the overall physiologically complex Tb architecture. Following the removal of isolated unsupported Tb structures, the remaining face groups were recombined, and the final rendered .stl model was exported.

### 2.5 Slicing and fabrication of fused deposition modelling and stereolithography 3D tibia models

The mouse tibia and femur were selected for further processing. For EB, the BioX™ bioprinter contained built-in slicer software. Rendered files were uploaded and visualised on the printer, determining the number of slices and geometrical information. No supports were added to the .stl model.

For FDM, the model was sliced for the printer to process. The rendered .stl model was uploaded into Ultimaker Cura. Following this, the model was re-sized within the edit feature (Table 1). Transform was selected, with coordinate space to local frame and uniform scaling selected to ensure translation of model volume in proportionate sizing to the method of 3D printing/bioprinting (Table 1), and the rotation of model was optionally applied to ensure even weight distribution and suitable centre of gravity of the model. The 3D printer stage and upper limits were input into the software, with the model fixed to the build plate. The infill density of the printer was selected, where 20% was used with a grid infill pattern and 0.8-mm minimum shell thickness. Two conditions of GCODE support were generated: connecting the model to the build plates only and connecting the model to itself and to the build plate. Supports were printed in sacrificial filament (Table 1). Selecting the slice function, the software generated the associated GCODE for the .stl model, enabling translation of the 3D model into a 3D printer system. FDM models were printed with a 0.4-mm nozzle width and a 0.2-mm layer height using the Prusa i3 MK2 (Prusa Research, Prague, Czech Republic) with multi-material V1 kit (Prusa Research) addition (Table 2).

**TABLE 1:** Original XYZ coordinates of mouse tibia, followed by translation of appropriate size for bio-printing, FDM, and SLA.

Model	X (mm)	Y (mm)	Z (mm)
Micro-CT STL output	2.55	2.48	1.51
Extrusion-based bioprinting	25.5	24.8	15.1
Filament deposition modelling	54	58	33
Stereolithography	54	58	33

Depending on the form of 3D printing/bioprinting and the material used, different total volumes can be achieved to replicate the original morphological complexity of the scanned object. Adaptive rendering, mm, and binary were used, with size translation using the scale function in Ultimaker Cura. Autodesk® Meshmixer models were translated by a 1,913% increase from the original model, whereas Autodesk models underwent a 98% reduction to generate equal-dimension models.

**TABLE 2:** Fused deposition modelling and stereolithography materials.

Printing material	Distributor and colour descriptor
PLA + filament 1.75 mm, 1 KG	eSUN, AC—Bone white
Siraya tech simple V2 water washable 3D printer resin, 1 KG	Siraya tech store—Grey V2
Water soluble support material, ø 1.75 mm, 500 g	Xioneer store—White

Lychee (Lychee, Bordeaux, France) was selected for the ease of use for slicing for SLA using the same parameters. Rendered models were uploaded into the software interface and scaled appropriately (Table 1) using the scale function. Automatic supports were generated per model, followed by exporting the model for 3D printing. SLA models were printed using the Nova3D Elfin 2 mono 3D printer (Nova3D, Shenzhen, China) with a 0.05-mm layer height, 1.5-s normal layer exposure, and 30-s bottom layer exposure prior to a final 2-min post cure at 405 nm. Printing success or failure was documented photographically and assessed qualitatively. Completion was defined as a finished 3D model being produced, with no major errors or excess spooling occurring during the printing process. FDM models with soluble supports underwent washing under warm water for 48 h to remove sacrificial filament.

### 2.6 3D model quantification

Micro-CT of the 3D-printed FDM and SLA models was conducted to compare the overall volume, surface area, and size with the original .stl mesh of detailed Autodesk® Fusion 360™ models. One model from each print setting was scanned using micro-CT (Skyscan 1272, Bruker) at 49.5 µm voxel, 0.1 mm aluminium filter, and 500 × 500 camera binning. Reconstructions used automatic misalignment correction, beam-hardening correction of 40%, and ring artefact reduction of 6. A 2-cm VOI of the original .stl mesh file was loaded into DragonFly 3D World software (version 2024.1), and the total volume (cm^3^), surface area (cm^2^), and height, length, and depth (cm) were calculated as a baseline measure. Subsequently, each 3D model for the individual print setting was loaded into DragonFly 3D World, the threshold was set at 63–255 greyscale, and a 2-cm section with 1-mm offset from the top of each model to match the same VOI of the mesh was quantified for the total volume (cm^3^), surface area (cm^2^), and height, length, and depth (cm), with n = 1/group.

### 2.7 Statistical analysis

Each mouse tibia (n = 3) was exported under every combination of meshing algorithm, encoding format, and unit. Statistical comparisons, therefore, evaluated repeated exports of the same underlying datasets under different computational conditions rather than independent biological replicates. Data were processed in Prism v8.1.1 (GraphPad software). All data were represented as individual data points, with the mean values displayed. When appropriate, Shapiro–Wilk normality testing was completed. Normally distributed data underwent Tukey’s multiple comparison test within each bone type. The mean file size and mesh error were compared between meshing algorithms, and statistical significance was accepted when *P* ≤ 0.05.

## 3 Results

### 3.1 *Ex vivo* bone visualisation and processing

**FIGURE 3:**
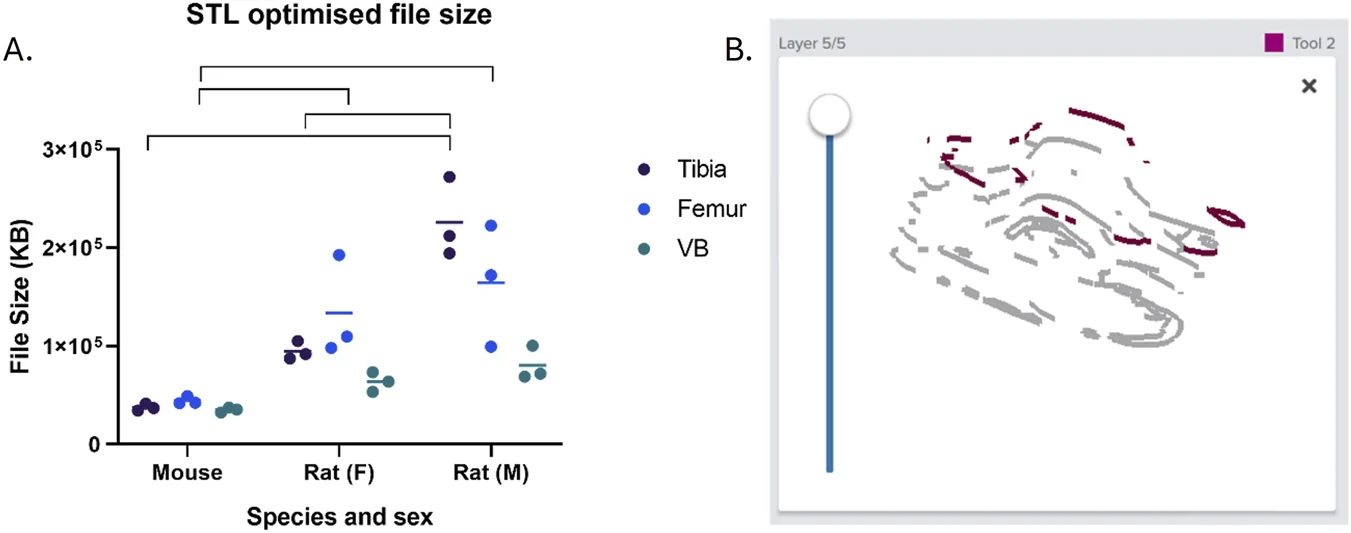
File size of STL output from CTAn of mouse and rat (F + M) tibia, femur, and VB. **(A)** 1.5-mm mouse VOI and 2-mm rat VOI were exported from CTAn with adaptive rendering in binary and mm units. Mean values of file size are represented with statistical significance defined (<0.05). n = 3. **(B)** Representative image depicting failed slicing on BioX interface of a mouse VB exported as .stl with a file size of > 55,000 KB.

**FIGURE 4:**
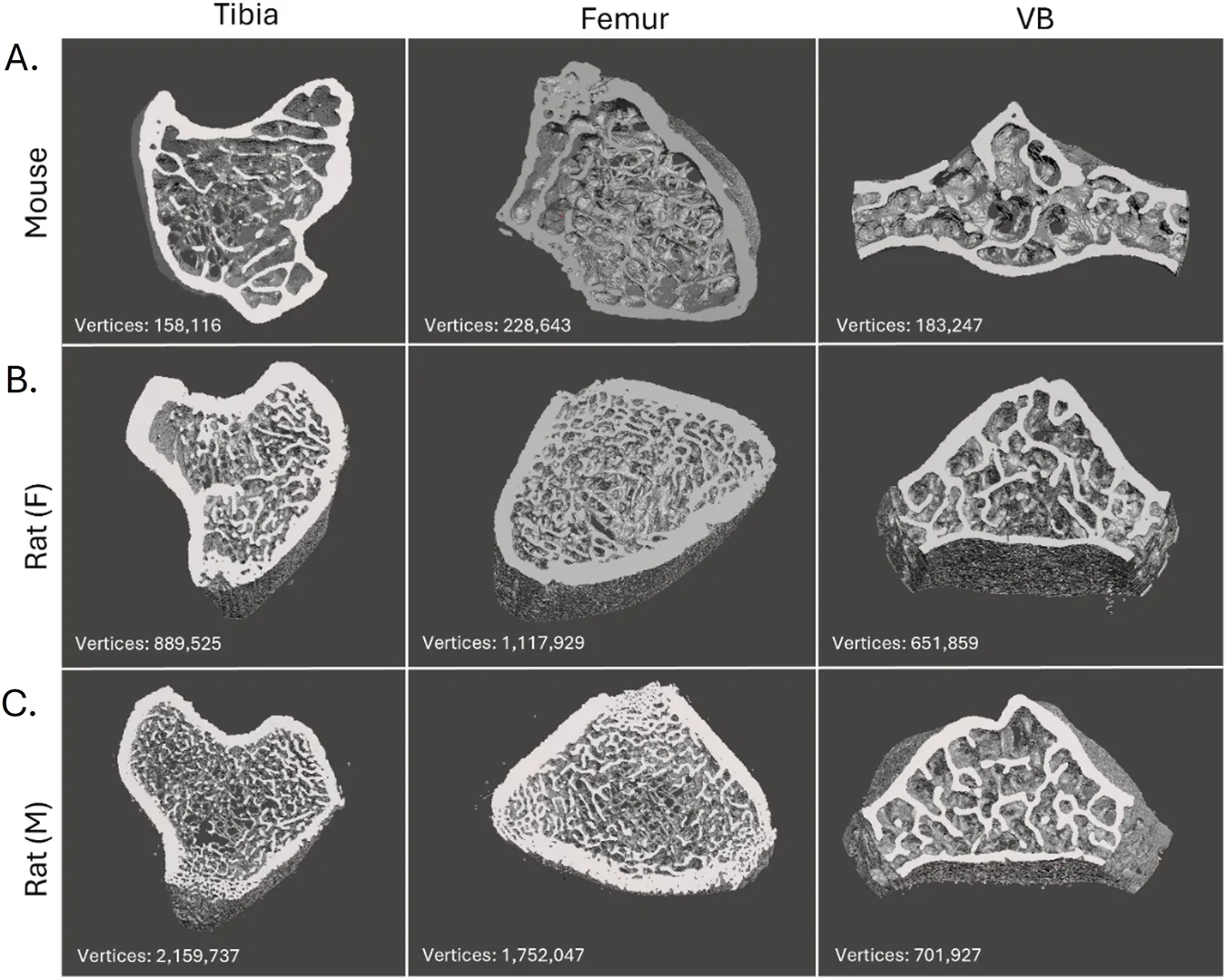
Tessellated 3D model of various mouse and rat bones using computer-assisted design (CAD) software (.stl). **(A)** Mouse (1.5 mm VOI), **(B)** female rat (2 mm VOI), and **(C)** male rat (2 mm VOI). Region of interest from a section of tibia, femur, and vertebral body (VB), including cortical bone (Cb) and trabecular bone (Tb), were exported as standard tessellation language (STL) files from micro-CT with adaptive rendering, +1 smoothing; binary and mm units were used to tessellate the object surface. STL models that were uploaded into CAD software units were used to tessellate the object surface. STL models were uploaded into CAD software Autodesk® Meshmixer for visualisation of models and vertex counts. n = 3; representative model shown.

All bone VOIs were exported directly from CTAn as .stl files without additional CAD rendering. File size comparisons between bone types showed no statistical difference in the VB between species; however, the femur and tibia exhibited a wide range of file sizes (Figure 3A). For the tibia, no significant difference in file size was noted between mouse and female (F) rat compared to that of male (M) rat (*P* < 0.0001 and 0.0002, respectively). In comparison, the file size of rat femur bones was statistically different between females and males [*P* = 0.0067 (F) and *P* = 0.0005 (M)] compared to that of mouse. The increase in complexity of bone structure for rats compared to that of mice was reflected in increased tessellation (Figure 4C) and correspondingly larger file size (Figure 3A). Furthermore, .stl files exceeding 36,000 KB failed to upload correctly into the BioX™ bioprinter interface (Figure 3B). Only 2 mouse VB models fell below the approximate threshold of 36,000 KB (Figure 3A), indicating a practical upper limit for successful file handling within this system.

All bone VOIs were uploaded into Autodesk® Meshmixer for visualisation and rendering. Consistent with file size trends (Figure 3A), VB across all species and sexes exhibited the lowest quantity of vertices among all bone types analysed. Across all model conditions, an increase in vertex count (Figure 4) corresponded to an increase in file size (Figure 3A). Rat (M + F) .stl models were successfully loaded; however, any subsequent manipulation within the CAD environment caused the failure of the computer system. The greater structural complexity of the rat bone, relative to mouse bones, was qualitatively evident and led to highly complex *in silico* 3D models (Figures 4B, C). Due to the complexity of the morphology and, therefore, the large .stl file size, rat-derived bones were excluded from further downstream analysis and processing. In contrast, mouse bones were successfully uploaded into the Autodesk® Meshmixer interface, reflecting their comparatively smaller dimension and reduced file sizes. The VB was additionally excluded from the study due to the thin layer of Cb around the Tb, which would have required manual wall thickening for successful rendering and manufacturing, thereby deviating from the native morphology and volume. Following these exclusions based on file size constraints and CAD processing limitations, only mouse tibia and femur models were retained for further analysis (Figure 4A).

### 3.2 Standard tessellation language algorithm, unit, and code optimisation on mouse tibia

**FIGURE 5:**
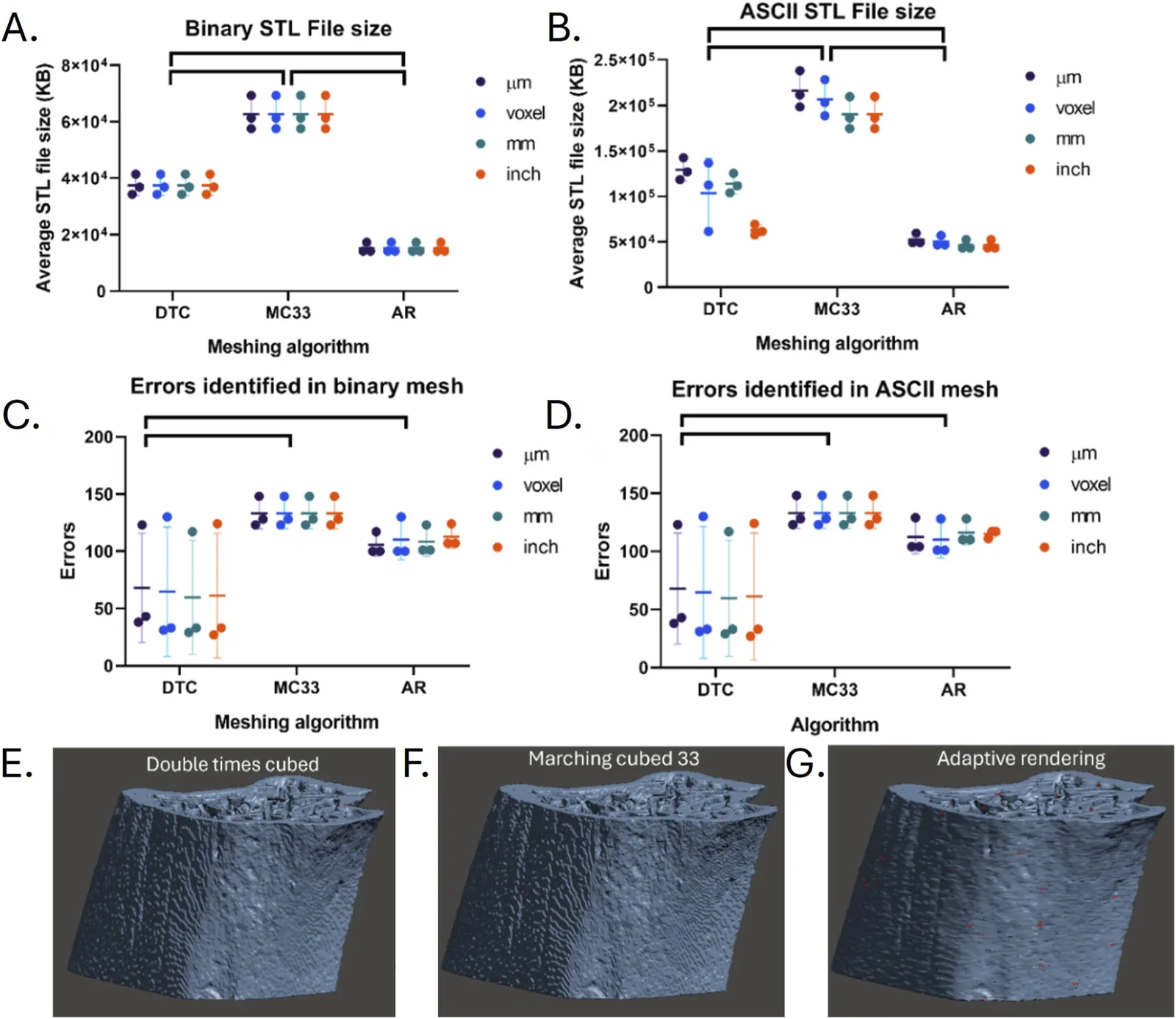
Section of mouse tibia exported into Autodesk® Meshmixer, inclusive of all potential 3D model tessellation conditions from CTAn to determine file size differences and mesh errors. A section of mouse tibia was imaged with micro-CT at 9-μm voxel pixelation and reconstructed with a binarisation of 0–0.12. A 1.5-mm VOI was exported as double times cubed (DTC), Marching Cubes 33 (MC33), and adaptive rendering (ATR) in either binary or ASCII code,and various units (um, voxel, mm, and inch). All generated STL models were exported into Autodesk Meshmixer; **(A)** binary file size, **(B)** ASCII file size, **(C)** errors identified in binary mesh, **(D)** errors identified in ASCII mesh, **(E)** double times cubed, **(F)** Marching Cubes 33, and **(G)** adaptive rendering with mesh errors highlighted by red pixels. n = 4; two-way ANOVA with Tukey’s multiple comparisons test against each meshing algorithm; *P* < 0.05.

Binary code consistently produced smaller file sizes across all export units compared to ASCII encoding (Figure 5). All algorithms produced significantly different total file size for both binary (*P* < 0.0001) (Figure 5A) and ASCII (*P* < 0.0001) (Figure 5B) export formats. These differences may reflect the level of structural complexity captured in the .stl model (Figures 5E–G). All algorithms produced mesh errors (Figures 5C,D). MC33 produced the highest frequency of errors compared to DTC (*P* < 0.0001) for both ASCII and binary formats, with ATR (*P* = 0.0022 and 0.005 for ASCII and binary, respectively). Although these meshing errors could be repaired in further rendering mesh-repair tools, such corrections may risk further deviation from the original imaged *ex vivo* bone model. Mesh errors were quantified only in Autodesk® Meshmixer, as Autodesk® Fusion 360™ did not provide equivalent metrics.

Each unit produced different output model dimensions intra-algorithm, as expected (Table 3). When matched units were compared across different algorithms, slight discrepancies in dimension were observed, highlighting differences in geometric complexity generated by changing algorithms. Despite variations in output units, each algorithm produced an equal frequency of triangles and vertices, indicating that the isosurface generation led to standardised geometric scaling while still adhering to standard tessellation rules. Considering the level of detail captured, particularly the surface morphometry and porosity (Figures 5E–G), it was necessary to assess whether this resolution could be reproduced using current 3D-printing techniques. Collectively, ATR with binary encoding and mm units provided the best balance between file size, reduced mesh errors, preservation of anatomical morphology, and compatibility with downstream CAD processing and AM techniques and was, therefore, selected for the remainder of the workflow.

**TABLE 3:** Average STL dimensions and tessellation values from different algorithms and units from C57 mouse tibia.

Algorithm	Unit	Size (mm)			Mesh	
		X	Y	Z	Triangles	Vertices
Double times cubed	*µm*	2,442.7 ± 12.5	1,505.9 ± 8.0	2,667.7 ± 13.2	768,563 ± 15,4320	383,890 ± 75,300
	*voxel*	271.0 ± 5.0	167.0 ± 3.5	296.0 ± 6.0	768,563 ± 15,4320	383,890 ± 75,300
	*mm*	2.4 ± 0.02	1.5 ± 0.01	2.7 ± 0.02	768,563 ± 15,4320	383,890 ± 75,300
	*inch*	0.1 ± 0.002	0.1 ± 0.002	0.1 ± 0.002	768,563 ± 75,300	383,890 ± 75,300
Marching Cubes 33	*µm*	2,447.9 ± 13.0	1,511.9 ± 8.5	2,678.9 ± 14.0	1,284,328 ± 25,350	641,610 ± 12,700
	*voxel*	180.4 ± 4.5	112.0 ± 2.5	201.0 ± 5.0	1,284,328 ± 25,350	641,610 ± 12,700
	*mm*	2.4 ± 0.02	1.5 ± 0.01	2.7 ± 0.02	1,284,328 ± 25,350	641,610 ± 12,700
	*inch*	0.1 ± 0.002	0.1 ± 0.002	0.1 ± 0.002	1,284,328 ± 25,350	641,610 ± 12,700
Adaptive rendering	*µm*	2,447.6 ± 11.5	1,511.9 ± 8.5	2,677.8 ± 13.0	317,613 ± 6,500	158,117 ± 3,200
	*voxel*	272.0 ± 5.0	168.0 ± 3.5	297.5 ± 6.0	317,621 ± 6,500	158,117 ± 3,200
	*mm*	2.4 ± 0.02	1.5 ± 0.01	2.7 ± 0.02	317,612 ± 6,500	158,116 ± 3,200
	*inch*	0.1 ± 0.002	0.1 ± 0.002	0.1 ± 0.002	317,619 ± 6,500	158,116 ± 3,200

VOI of 1.5 mm (Y-axis) was subjected to different algorithms and unit options in CTAn and uploaded into Autodesk® Meshmixer to visualise XYZ dimensions and total mesh tessellation of the STL files. n = 3; mean values are reported; ASCII and binary produced equal values.

### 3.3 Computer-assisted design rendering

**FIGURE 6:**
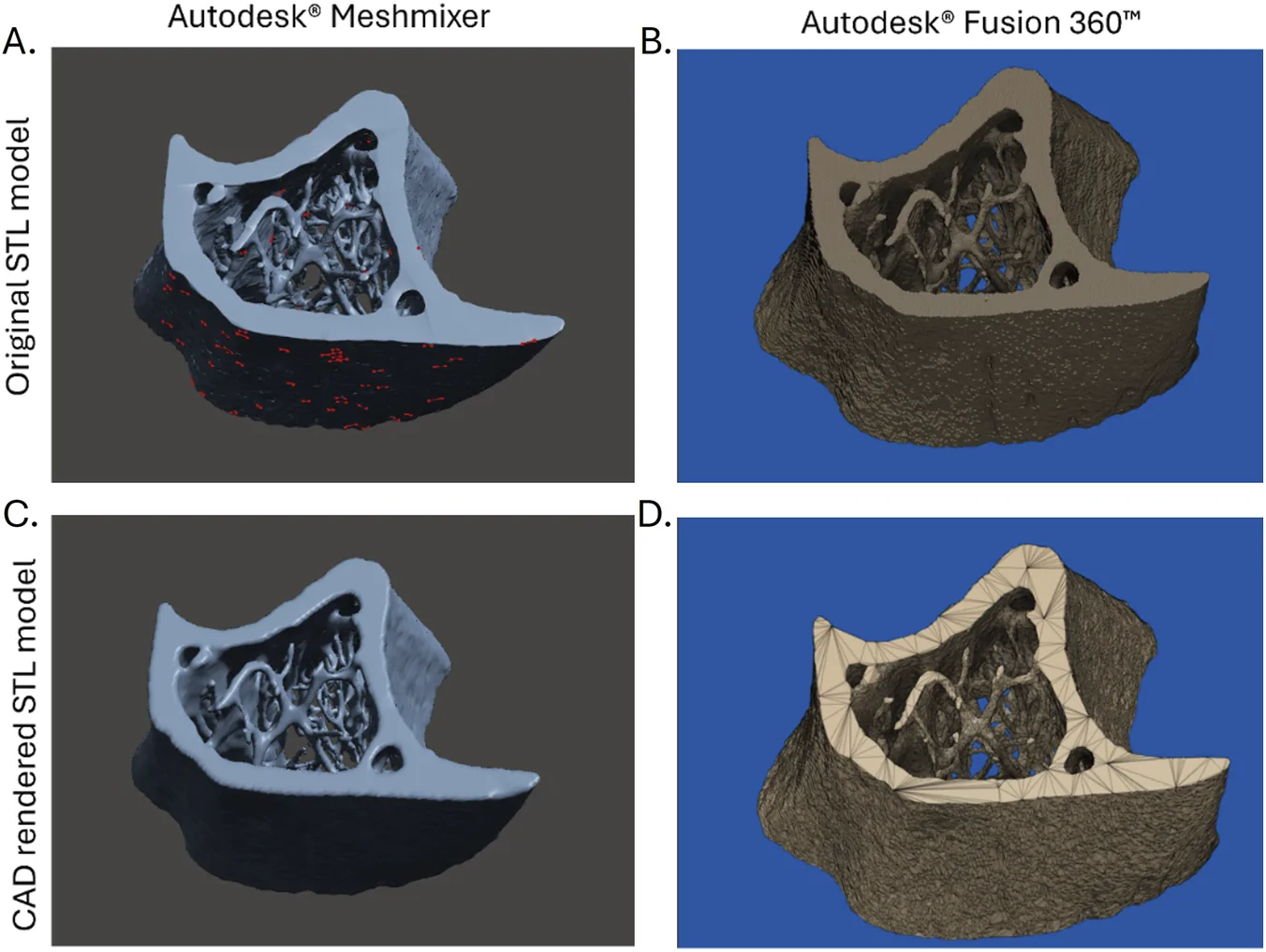
Mouse tibia mesh in Autodesk® Meshmixer and Autodesk® Fusion 360™ illustrating differences in surface topography. The original .stl model is shown in both interfaces **(A,B)**, with the rendered output .stl from Meshmixer **(C)** and Fusion 360™ **(D)**.

Reduction of tessellation decreased the file size while simplifying the surface geometry to a level compatible with the resolution limits of printing technologies and material (Guida et al., 2024). Different CAD software packages utilize distinct tessellation algorithms, which are unknown to the average user. This leads to variations in mesh structure and surface geometry arising solely from the choice of software (Figures 6A,B). Although rendering options are varied between CAD software, reduction of tessellation, manifold correction, and mesh repair are essential processes to improve printability and minimise fabrication errors. However, every modification applied during rendering has the potential to alter the geometry of the original micro-CT model (Figures 6C,D). Consequently, rendering decisions were limited to those considered necessary to facilitate successful downstream processing and fabrication while minimising deviation from the original bone morphology.

**FIGURE 7:**
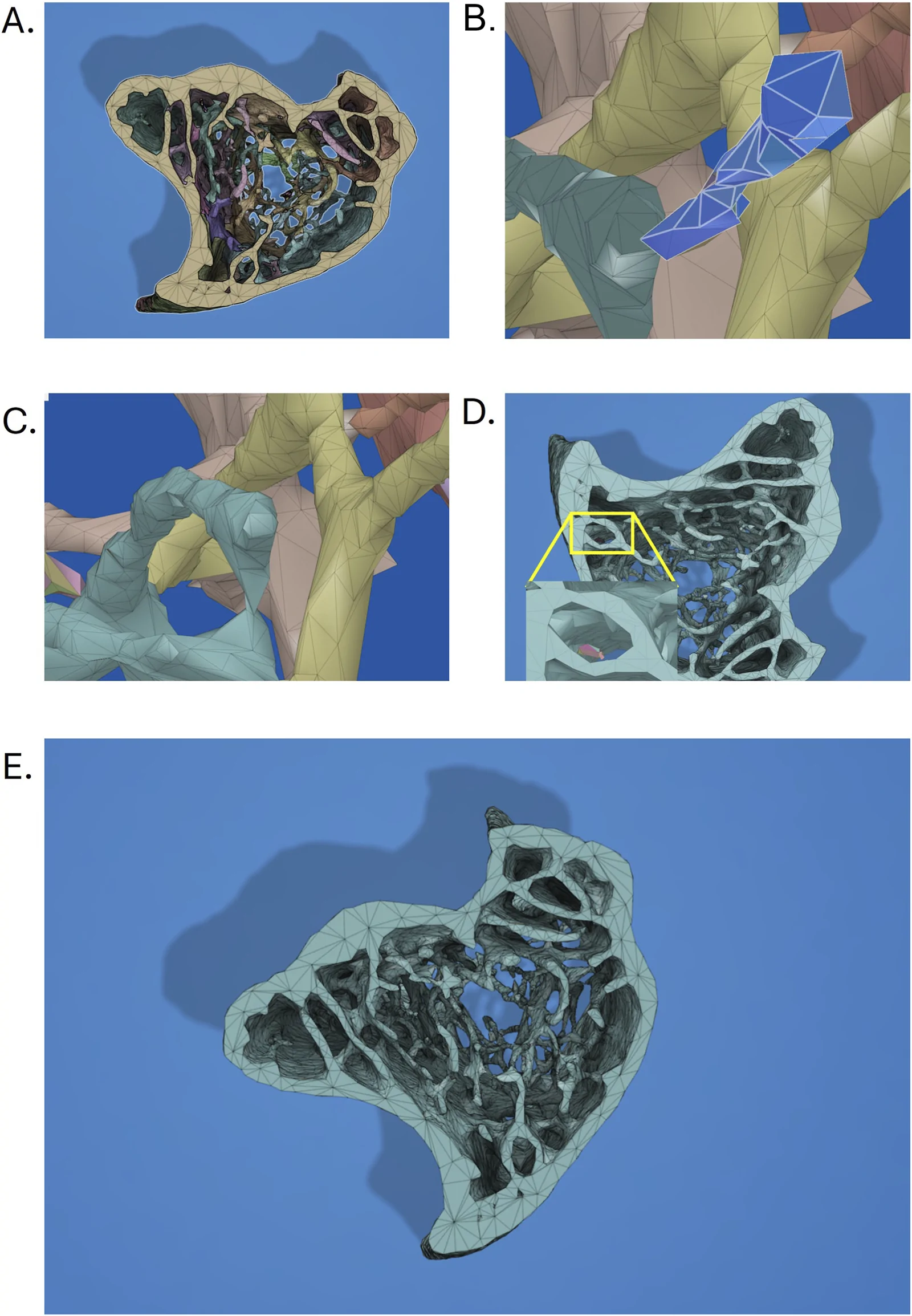
Identification and removal of unconnected trabecular structures in Autodesk Fusion 360™. A 1.5-mm VOI of mouse tibia was exported as an STL model with an adaptive rendering algorithm, binary format, and mm unit. Following the basic rendering of a 20% reduction in tessellation and repairing mesh in Fusion 360™, a detailed analysis can be completed to improve future printability by removing unconnected biological structures. **(A)** Mouse tibia with face-groups generated to visualise tessellation in different planes; **(B)** enlarged area of Tb structures, with unconnected Tb manually identified (blue); **(C)** unconnected Tb structure manually removed; **(D)** identification and removal of small unconnected bone fragments, where the yellow box represents the area shown in the manual zoom; **(E)** face-groups combined with all non-supported structures removed from the model.

The complex architecture of biological bone is naturally supported by surrounding tissues, including bone marrow. During micro-CT segmentation, these supporting tissues were removed to isolate mineralised bone, and a manually defined VOI was selected, which occasionally led to individual Tb structures becoming disconnected from the main Cb and Tb network. To assess if unsupported Tb would interfere with printing success, unconnected sections of Tb were identified using CAD face groups and removed in a deliberate engineering compromise between manufacturability, print fidelity, and preservation of biologically relevant architecture (Figures 7A–D). Face groups organise connected mesh structures according to standard tessellation rules, allowing isolated unsupported structures to be identified as separate components. Retaining these isolated structures was considered likely to increase the probability of printing failure by generating unsupported features during fabrication.

### 3.4 Slicing the rendered 3D *ex vivo* bone models

**FIGURE 8:**
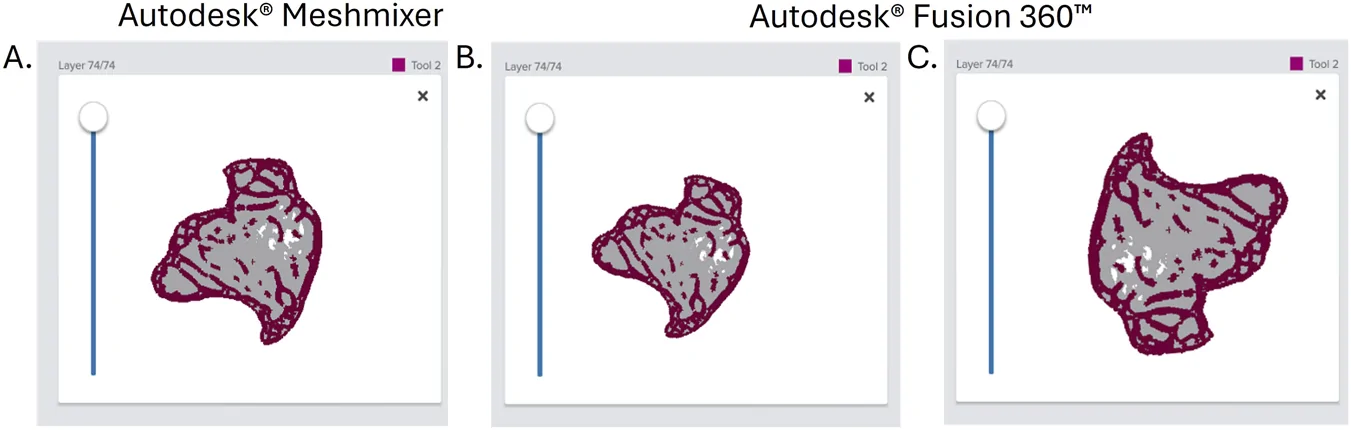
Mouse tibia that was STL-rendered and uploaded to BioX bioprinter. The *ex vivo* bone model consists of 76 layers, with the current layer visualised in red. The model’s dimensions are summarised in Table 3. **(A)** Meshmixer basic, **(B)** Fusion 360™ basic, and **(C)** Fusion 360™ detailed.

Slicer software translates a 3D model into 2D planar XYZ information with layer-wise Z information in the form of G-code for 3D printing. The bioprinter used in this study, the BioX™, contains a built-in slicer software application. All rendered .stl models from Autodesk® Meshmixer and Autodesk® Fusion 360™ were uploaded into the interface and produced identical sliced outputs, each consisting of 76 layers (Figure 8).

**FIGURE 9:**
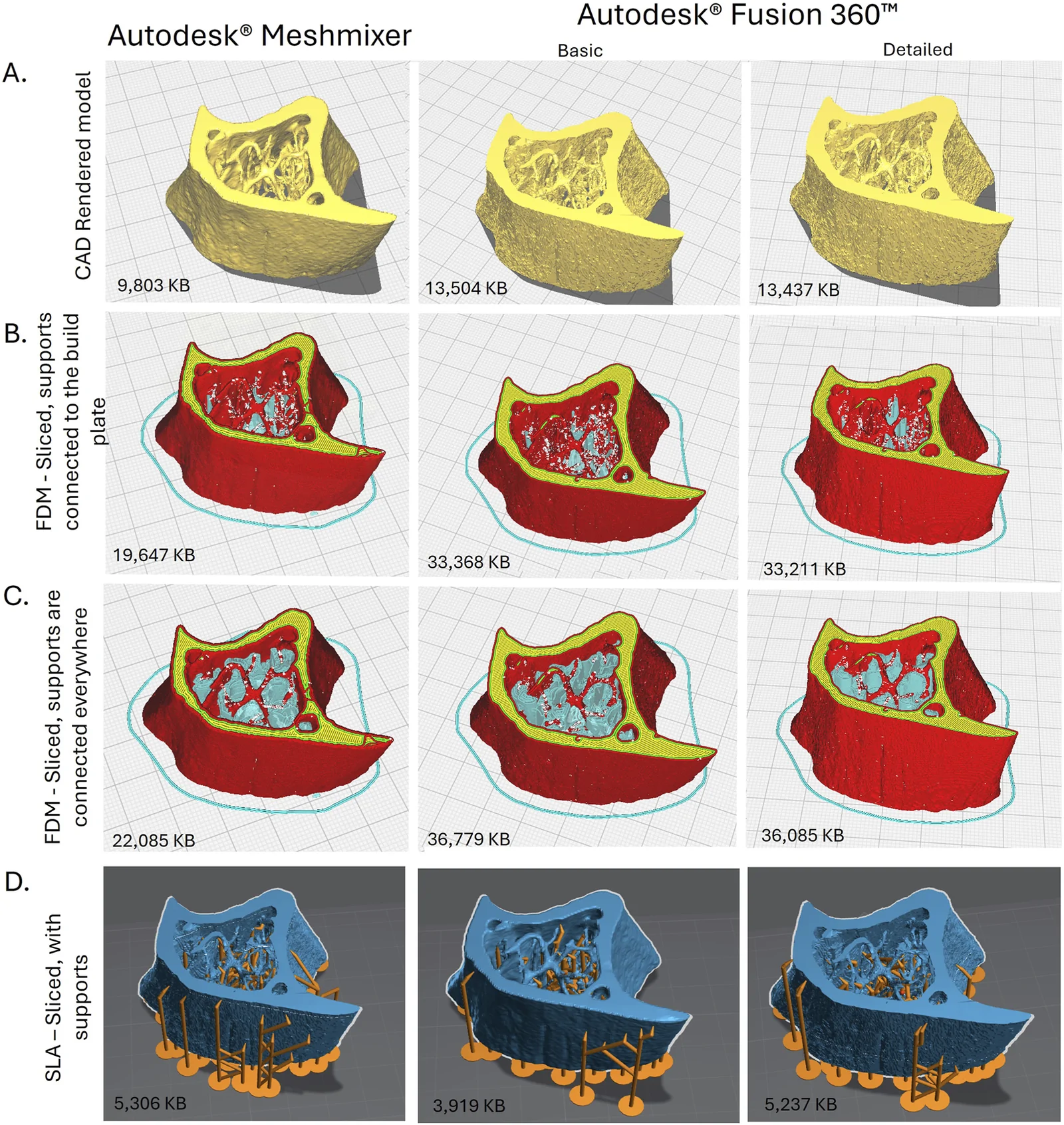
STL models rendered in Autodesk® Meshmixer and Fusion 360™, with FDM models sliced in Ultimaker Cura 5.9.0 and SLA sliced in Lychee. Rendered models are uploaded into either Cura or Lychee. The Cura interface models underwent either supports built to the build plate only or everywhere within the 3D model. All models were translated to the same coordinates; X: 54 mm, Y: 58 mm, and Z: 33 mm. File size can be visualised in each tile for the respective model throughout the process. **(A)** CAD-rendered model set. **(B)** Sliced models with supports connected to build plate only for fused deposition modelling. **(C)** Sliced models with supports connected everywhere for fused deposition modelling. **(D)** Sliced models with supports for stereolithography. Colour code for Cura: inner wall (green), top (yellow), infill (orange), shell (red), and supports (blue). Colour code for Lychee: 3D model (blue) and supports (orange). n = 1.

For FDM, supports were added in two conditions: build plate connection only (Figure 9B) and everywhere (Figure 9C). SLA-generated supports connected to the build plate and inner unsupported structures (Figure 9D). Autodesk® Meshmixer produced a lower file size compared to that by Autodesk® Fusion 360™; however, FDM slicing increased the file size of the .stl models (Figures 9B,C), compared to SLA slicing, which reduced the overall file size (Figure 9D). For each model, the supports generated highlighted the variance in the different rendering processes by the location of supports. Across all FDM conditions inclusive of supports, the file size was greatly increased compared to the original CAD rendered model (Figures 9A–C).

### 3.5 Fused deposition modelling and stereolithography 3D models

**FIGURE 10:**
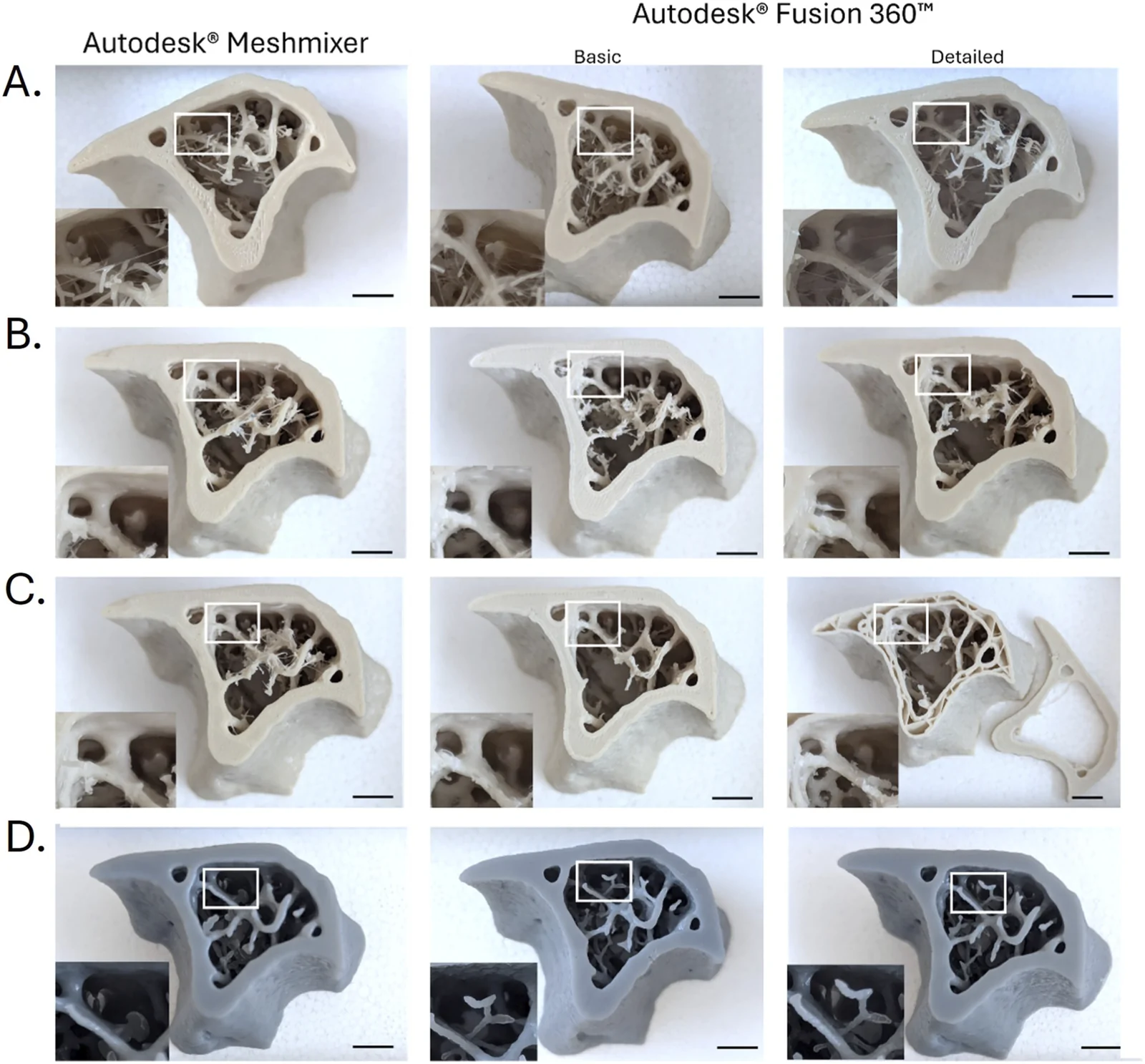
Fused deposition and stereolithography of *ex vivo* murine tibia bone 3D-printed models. Rendered and sliced murine tibia were printed by FDM with polylactic acid polymer, and SLA with resin; **(A)** FDM, no structural supports; **(B)** FDM, structural supports connected to build plate only; **(C)** FDM, structural supports everywhere; **(D)** SLA, structural supports removed. Representative images of model; n = 1 of each CAD rendered condition; scale bar represents 1 cm*.*

FDM 3D tibial models were successfully fabricated without supports, producing grossly representative models inclusive of both Cb and Tb structures (Figure 10A); however, the models exhibited filament stringing, leading to qualitative deviation from the original rendered .stl models (Figures 6, 7). These deviations were most evident within the Tb region, where filament stringing and over-extrusion of filament were observed, potentially caused by frequent stop/start motion required to fabricate the complex Tb architecture. Individual print layers remained visible, and loss of Tb definition together with structural discrepancies was observed across the three different CAD software programs (Figure 10). The removal of isolated unsupported Tb structures (Figure 9) was essential to achieve a successful print, as all conditions managed to produce printable 3D models without FDM printing errors. To improve printing accuracy, particularly for the overhanging Tb structures, printing supports were applied in two configurations: build plate only and everywhere (Supplementary 1). Following removal of build-plate-only supports, the underlying architecture of the 3D models was revealed (Figure 10B).

Across the three CAD fabrication methods (Figures 6, 7), no major fabrication defects were observed within the Cb shell; however, individual filament layers remained visible. In contrast, Tb structures consistently exhibited filament over-extrusion, stringing, and reduced architectural definition across all conditions (Figures 10A–C), leading to qualitative deviation from the original .stl *ex vivo* mouse tibia (Figure 6A). The addition of build-plate-only supports did not produce a qualitative improvement in the preservation of Tb architecture compared to that with unsupported prints (Figures 10A,B, respectively). The use of full sacrificial supports everywhere led to additional fabrication failures during FDM printing. In the Autodesk® Fusion 360™ model, the Cb shell exhibited gaps between filament layers, leading to structural failure during support removal. Similar gaps were also observed in the Cb shell across both Autodesk® Fusion 360™ conditions (Figure 10C). In addition, Tb structures were reduced in models printed with full supports (Figure 10C) compared to those with build plate only (Figure 10B) and no supports (Figure 10A).

Resin-printed models produced *via* SLA were printed with minimal structural supports. The models featured greater preservation of Cb surface detail relative to the original input micro-CT .stl model, and defined Tb overhanging structures were also noted. Differences between the three CAD models were observed within the Tb region, particularly between the Autodesk® Meshmixer and Autodesk® Fusion 360™ models (Figure 10D). Compared to the FDM-printed models (Figures 10A–C), SLA models qualitatively demonstrated improved preservation of Cb surface texture and Tb architecture, with reduced visible fabrication artefacts (Figure 10D). These observations were based on qualitative visual assessment and require quantitative validation to determine the extent of geometric preservation. Collectively, these findings demonstrate that FDM was capable of producing grossly representative bone models but was limited in preserving fine Tb architecture under the fabrication conditions investigated.

### 3.6 Quantitative 3D analysis

**FIGURE 11:**
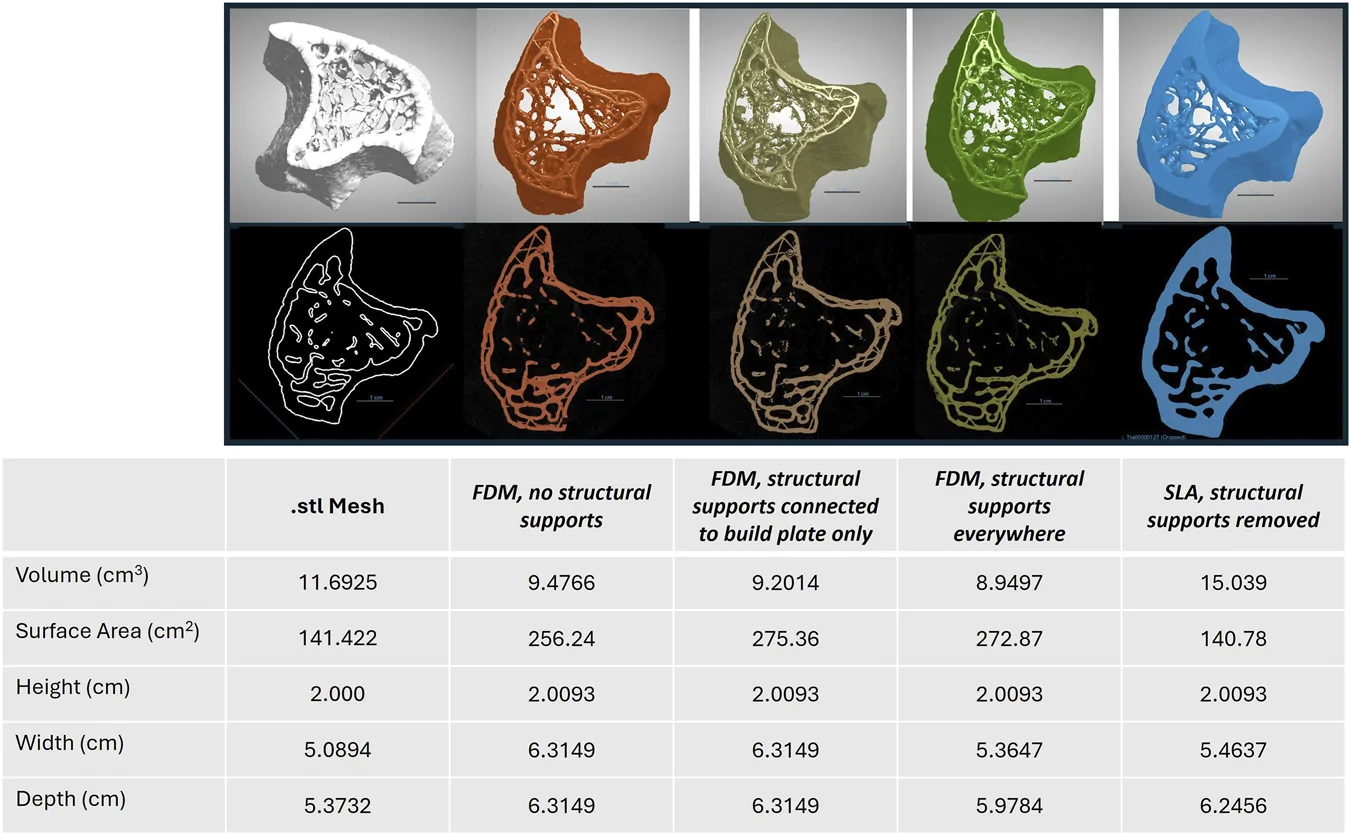
3D model quantification compared to original .stl mesh. Autodesk® Fusion 360™ detailed .stl mesh and 3D-printed models used. Volume (cm^3^) represents the amount of 3D space that is occupied, whereas surface area (cm^2^) represents the total area of the outer surface of the model. n = 1; scale bar represents 1 cm.

As shown in Figure 11, due to the nature of FDM, all three models demonstrated a larger overall surface area (cm^2^) as a consequence of the hollow 3D frame. The hollow nature of this framework could also explain the overall reduction in the total model volume (cm^3^) compared to the VOI of the original .stl model, as there is less material in-fill between the layers. However, the SLA model was solid and had a larger overall volume (cm^3^) than the .stl model. This may be accounted for by the increase in model depth, at nearly 1 cm deeper than the .stl model. It is noted that the overall surface area of the SLA model was in line with that of the .stl model despite an increase in the total model volume (cm^3^). The preservation of surface area despite differences in the total volume indicates that the overall geometric mesh of the printed model was retained, while alterations to internal architecture and/or material fabrication may have contributed to differences in volumetric measurements.

## 4 Discussion

The workflow presented here aimed to outline a proof-of-concept methodology for scaling high-resolution micro-CT images of small model organism *ex vivo* bone VOI into .stl files suitable for AM while balancing preservation of physiologically relevant bone microarchitecture with the computational and manufacturing constraints of current CAD software and 3D-printing technologies. Furthermore, the methodological process and the considerations to translate biological high-resolution imaging into physical 3D models instead focus either on simple lattice structures (Fraser et al., 2024; Luttrell et al., 2019; Lv et al., 2024) or macro-porous architecture of biomaterials (Ahmed, 2015; Jiao et al., 2023; Martinez-Garcia et al., 2022). As far as the researchers are aware, no research explores the process of translating scaled physiologically accurate Tb and Cb into a large 3D printable model using different forms of AM.

The rendering process of the 3D CAD model should be tailored to the intended application, printing technique, and materials used within the fabrication process (Capellini et al., 2024; Qu et al., 2024; Qu et al., 2024; Qu et al., 2024). Murine bone was selected as a biologically complex proof-of-concept model to represent the fine-scale Cb and Tb architecture to provide a stringent test of the micro-CT to AM workflow. FDM and SLA murine tibiae, inclusive of Cb and Tb, were successfully fabricated, with different processing parameters explored for each manufacturing approach. Throughout the workflow, methodological decisions were tailored to each AM process to minimise deviation from the original *ex vivo* micro-CT model while achieving successful fabrication of the representative bone morphology. The method presented here is intended as a research framework for generating anatomically informed 3D bone models and exploring the considerations required within this process. Potential future applications include disease modelling, anatomical education, and the development of patient-specific manufacturing workflows (Capellini et al., 2024; Tripodi et al., 2020). However, translation towards applications such as personalised prosthetics will require further validation, including quantitative assessment of geometric fidelity, mechanical characterisation, biological evaluation, and clinical investigation. Current challenges for patient-specific prosthetics include restoring the complex architecture of bone and its mechanical and biological function (Qu et al., 2024; Qu et al., 2024; Qu et al., 2024; Zhao et al., 2024). The workflow presented here represents an initial framework for addressing the geometric components of this challenge by enabling the fabrication of anatomically informed bone models while recognising that substantial biological, mechanical, and clinical validation is necessary before translational application.

The observation of deviation by different CAD rendering processes was supported experimentally by Abad-Coronel (2023); Kamio (2020), and Rungrojwittayakul (2020) and mathematically explored by Hällgren (2016). The researchers compared the resulting tessellation and 3D prints from different CAD systems, suggesting that the tessellated surface differences between the systems is due to the widely-used .stl file format and the translation of the mesh boundaries between different software algorithms (Abad-Coronel et al., 2023; Hällgren et al., 2016; Kamio et al., 2020; Rungrojwittayakul et al., 2020). The .stl file format follows an unstructured tessellation, highlighted in the Cb tessellated structure in Autodesk® Fusion 360™, following inconsistent geometric rules that facilitate curvature in the mesh. As a result, it may perhaps assist in the error production of the biological structures’ mesh due to the imperfect geometric textured surface. The meshing algorithm Autodesk® Fusion 360™ states in the software configuration is adaptive meshing, and it is unstated for Autodesk® Meshmixer. It could be suggested that different CAD software applications identify mesh errors with different boundaries.

Two methods of large-scale AM were explored to visualise the impact of different fabrication methods on the 3D models despite equal 3D .stl input. Both qualitatively and quantitively, SLA produced models with greater preservation of architectural detail and fewer visible fabrication artefacts than FDM under the conditions investigated. Methods to compare the 3D-printed models to the original input include surface deviation analysis for a mesh-to-mesh comparison by re-imaging the output 3D model and remapping for geometric deviation (Jadayel and Khameneifar, 2020; Qu et al., 2024; Qu et al., 2024; Qu et al., 2024). Alternatively, dimensional accuracy measurements can be taken from the physical 3D model’s landmarks and compared computationally to the CAD model within software (Wendo et al., 2025) such as Autodesk® Fusion 360™ measuring function. Quantitative surface deviation analysis represents an important next step in validating the geometric fidelity of the workflow (Abbasi et al., 2025). Future work would aim to incorporate further deviation analysis and assess high-resolution printers to explore deviation without the limitation of the printer type.

All FDM models exhibited filament over-extrusion for Tb architecture. Similar conclusions were drawn by Anadioti (2022), who reported significant deviations in all dimensions for FDM dental implants compared to the original CAD input, whereas SLA only produced deviations in one dimension. Anadioti (2022) used a very simplified block model, with no intricate architecture (Anadioti et al., 2022). In further detail, McMenamin (2014) reported that for anatomy printing with FDM, any structure over 10 mm was accurate in size, with an average error of 1.25% across all filament deposition above this size. Below 10 mm, error increased to 14.52%, and below 4 mm, filament had an error of 17.92% (McMenamin et al., 2014). Technique and resolution for fabrication *via* FDM has improved in the last 10 years. Ahn (2024) printed a filament diameter of 1.75 mm accurately, whereas Behseresht (2024) developed a closed-loop control printing system that self-recognises printing deviations and self-corrects during the printing process. Improvements continue to be made for SLA as well (Iftekar et al., 2023). Msallem (2024) discussed the significant advancements of the fabrication method in recent years with the high accuracy achieved with new SLA technology when printing an anatomical skull and mandible (Msallem et al., 2024) compared to a previously published study printing the same model with different technology (Msallem et al., 2020).

## 5 Conclusion

In this study, we present a proof-of-concept framework for transforming *ex vivo* murine bone data sets, inclusive of Cb and Tb microarchitecture, into anatomically informed .stl models while balancing preservation of bone morphology with the computational and manufacturing constraints of current software and AM technologies. Optimised export conditions of adaptive rendering, binary encoding and millimetre units provided a practical standardised workflow for generating CAD-compatible models from CTAn data sets. Controlled rendering was shown to be essential for reducing computational complexity and facilitating successful fabrication while minimising deviation from the original micro-CT geometry. The workflow provides a practical and insightful framework for biomedical research involving AM techniques. However, the biological complexity of bone frequently generated excessive mesh isoforms due to the high-resolution scanning parameters, making file size a key computational constraint. Under the conditions investigated, a practical file size limit of approximately 50,000 KB provided an appropriate balance between the preservation of physiologically relevant morphology and compatibility with downstream CAD processing. Different CAD algorithms and rendering operations inevitably modify the original micro-CT geometry. Consequently, rendering should be limited to the essential operations required to achieve complete tessellation, appropriate scaling, and successful fabrication while minimising geometric deviation from the original dataset.

## Data Availability

The datasets presented in this study can be found in online repositories. The names of the repository/repositories and accession number(s) can be found below: https://shura.shu.ac.uk/36872/.
